# HEALTH CARE AND SOCIAL CARE AMONG FRAIL OLDER ADULTS IN SWEDEN: A REGISTRY-BASED STUDY

**DOI:** 10.1093/geroni/igz038.1826

**Published:** 2019-11-08

**Authors:** Marie Ernsth Bravell, Deborah G Finkel, Linda Johansson

**Affiliations:** 1 Institute of gerontology, School of Health and Welfare, Jönköping University, Jönköping, Sweden; 2 Indiana University Southeast, New Albany, Indiana, United States

## Abstract

In 2010 the Swedish government made a large investment to improve the quality of care for the “frailest older adults” but there have been few follow-ups of the care of this group. The aim of this study is to therefore describe use of care over the time in 2010-2014. Methods: In 2014, 9 National Quality Registries, 3 Care Registries (drug-, patient and death-registers) and the registry for care and social services for older persons (SOL) were individually matched to an older population in the Swedish Twin Registry (n≈45000). Identification of the “frailest older adults” was achieved via SOL and the Patient Registry. Results: 280 persons were identified as “frailest” in 2010 these were followed over time. About two thirds (60,7%) were women, mean age: 81.2±7.7, about one third (35%) lived in a nursing home, one third (34%) had more than three hospitals stays, almost three quarters (72.5%) had more than 19 hospital days and almost one third (28%) had more than seven outpatient care visits. By the end of 2012, 119 persons (42.5%) were deceased. Among those alive (n=161) 42 persons did not receive any inpatient care between 2010-2012, but more than 85% received outpatient care and 44.1% were living in nursing homes. In 2014, 90 persons (32.1%) were still alive and half of them lived in a nursing home. In conclusion, over time the use of care as well as the risk of mortality was high confirming the need of further improvement of the care for these persons.

